# Increasing MinD’s Membrane Affinity Yields Standing Wave Oscillations and Functional Gradients on Flat Membranes

**DOI:** 10.1021/acssynbio.0c00604

**Published:** 2021-04-21

**Authors:** Simon Kretschmer, Tamara Heermann, Andrea Tassinari, Philipp Glock, Petra Schwille

**Affiliations:** † Department of Cellular and Molecular Biophysics, Max-Planck-Institute of Biochemistry, Am Klopferspitz 18, 82152 Martinsried, Germany; ‡ Current affiliation: Department of Bioengineering and Therapeutic Sciences, University of California San Francisco, San Francisco, California 94158, United States

**Keywords:** pattern formation, pattern engineering, self-organization, *in vitro* reconstitution, reaction-diffusion system, Min proteins

## Abstract

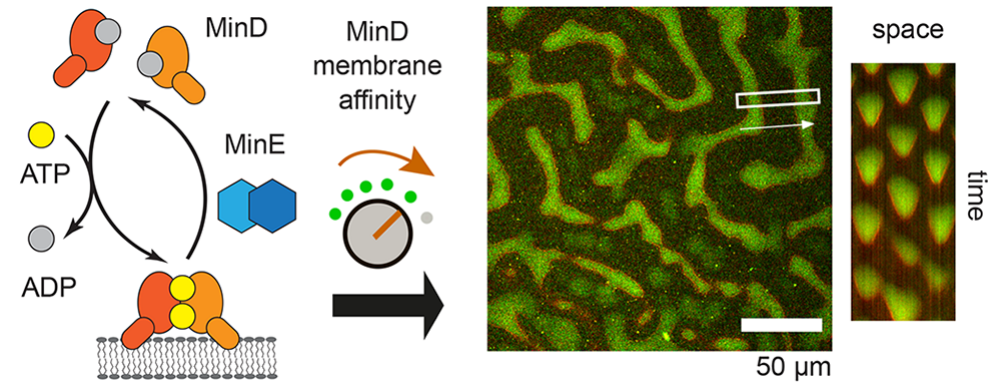

The formation of large-scale patterns through molecular self-organization is a basic principle of life. Accordingly, the engineering of protein patterns and gradients is of prime relevance for synthetic biology. As a paradigm for such pattern formation, the bacterial MinDE protein system is based on self-organization of the ATPase MinD and ATPase-activating protein MinE on lipid membranes. Min patterns can be tightly regulated by tuning physical or biochemical parameters. Among the biochemically engineerable modules, MinD’s membrane targeting sequence, despite being a key regulating element, has received little attention. Here we attempt to engineer patterns by modulating the membrane affinity of MinD. Unlike the traveling waves or stationary patterns commonly observed *in vitro* on flat supported membranes, standing-wave oscillations emerge upon elongating MinD’s membrane targeting sequence via rationally guided mutagenesis. These patterns are capable of forming gradients and thereby spatially target co-reconstituted downstream proteins, highlighting their functional potential in designing new life-like systems.

Living systems rely on periodic cycles in the expression or localization of proteins to regulate diverse processes such as metabolism,^1^ cell division,^2^ or development.^3^ Accordingly, the engineering of oscillating processes has been a major focus of synthetic biology. Temporal oscillations of protein levels have successfully been realized both in cellular^4−6^ and cell-free systems^7^ through synthetic gene networks. On the other hand, the engineering of protein oscillations in both space and time, as well as the resulting formation of defined spatiotemporal patterns has so far proven to be less readily accessible. In part, this is due to a limited understanding of naturally occurring pattern-forming protein systems and their molecular-level control parameters.

The *E. coli* Min system has become a paradigm for self-organized protein pattern formation^8,9^ and is therefore an ideal experimental testing ground for pattern engineering. In particular, the combination of protein-level mutagenesis and pattern-level analysis can be used to reveal how the tuning of molecular interactions translates into changes in the emergent properties of large-scale patterns. Min protein self-organization only requires the two proteins MinD and MinE, a lipid membrane as a catalytic matrix for self-organization, and ATP as a source of chemical energy.^10^ The use of the Min system as a model for protein pattern formation is facilitated by various experimental strategies for reconstituting Min patterns *in vitro* (for the major setups, see the recent review by Ramm et al.^8^). Moreover, theoretical frameworks have been developed to mathematically model Min protein self-organization.^9^

*In vivo*, the MinCDE system regulates cell division via a time-averaged concentration gradient of MinC, an inhibitor of the essential division protein FtsZ.^11^ This gradient displays maxima at the cell poles and a minimum at midcell and arises from pole-to-pole oscillations of the deviant WalkerA-type ATPase MinD and its ATPase activating protein MinE.^12,13^ On a mechanistic level, Min patterns emerge from the ATP-dependent cycling of MinD and MinE between the lipid membrane and the cytosolic bulk^10,14^ (Figure 1). MinD interacts with the membrane via a C-terminal membrane targeting sequence (MTS) in the form of an amphipathic helix^15−17^ (Figure 2a). The affinity of one copy of this MTS has been reported to be too weak for significant MinD binding to the membrane.^15,18^ However, upon ATP-dependent homodimerization, MinD gains sufficient affinity to accumulate on the membrane, which occurs in a cooperative fashion^15,18−22^ (Figure 1). Membrane-bound MinD then recruits MinE, which thereby undergoes a conformational switch from a latent state to a reactive state that can stimulate MinD’s ATPase activity^23,24^ (Figure 1). Upon MinE-induced ATP hydrolysis, MinD monomers detach from the membrane in the ADP-bound state and then undergo further cycles of Min protein accumulation on the membrane^18^ (Figure 1). The collective attachment and detachment of Min proteins to and from the membrane can give rise to a variety of different patterns depending on the experimental conditions.^8^ On a flat membrane, in the absence of flow in the bulk, prominent patterns include traveling waves and a variety of stationary (or quasi-static) patterns.^10,25^ In a flow-cell setup^26^ or via optical entrainment using a phototswitchable MinE peptide,^27^ standing-wave-like pattens were observed.^26^ Lastly, pole-to-pole oscillations and functional gradient formation can be reconstituted under confinement conditions mimicking the cellular environment.^28−30^

**Figure 1:**
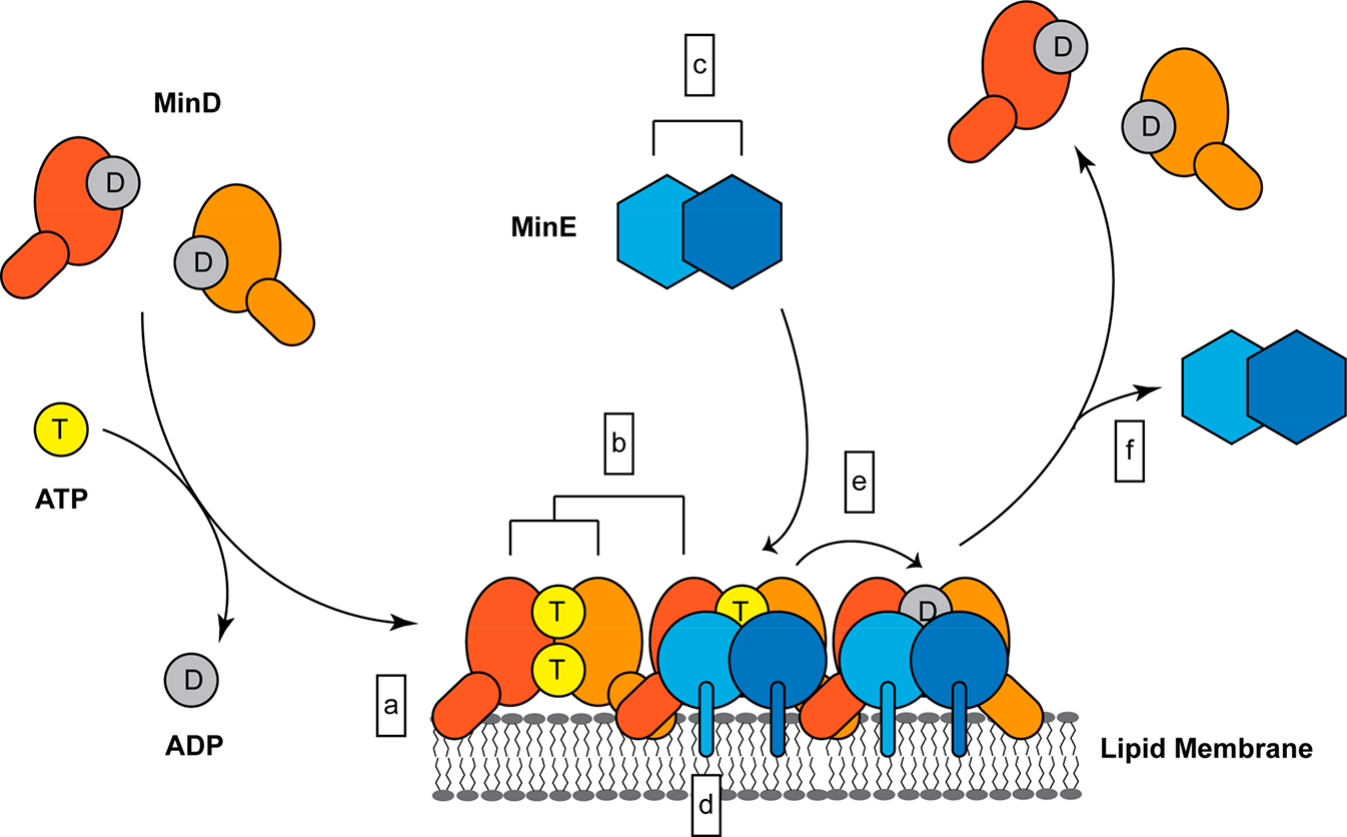
Functional modules implicated in the formation and regulation of Min protein patterns and references for their *in vitro* characterization: (a) MinD membrane interaction, this work; (b) MinD dimerization and higher-order interactions;^19,21^ (c) MinE dimerization;^31^ (d) MinE membrane interaction;^26,31,32^ (e) stimulation of MinD’s ATPase activity by MinE;^31,33^ (f) MinE’s conformational switch from a reactive to a latent state.^31,34^

**Figure 2:**
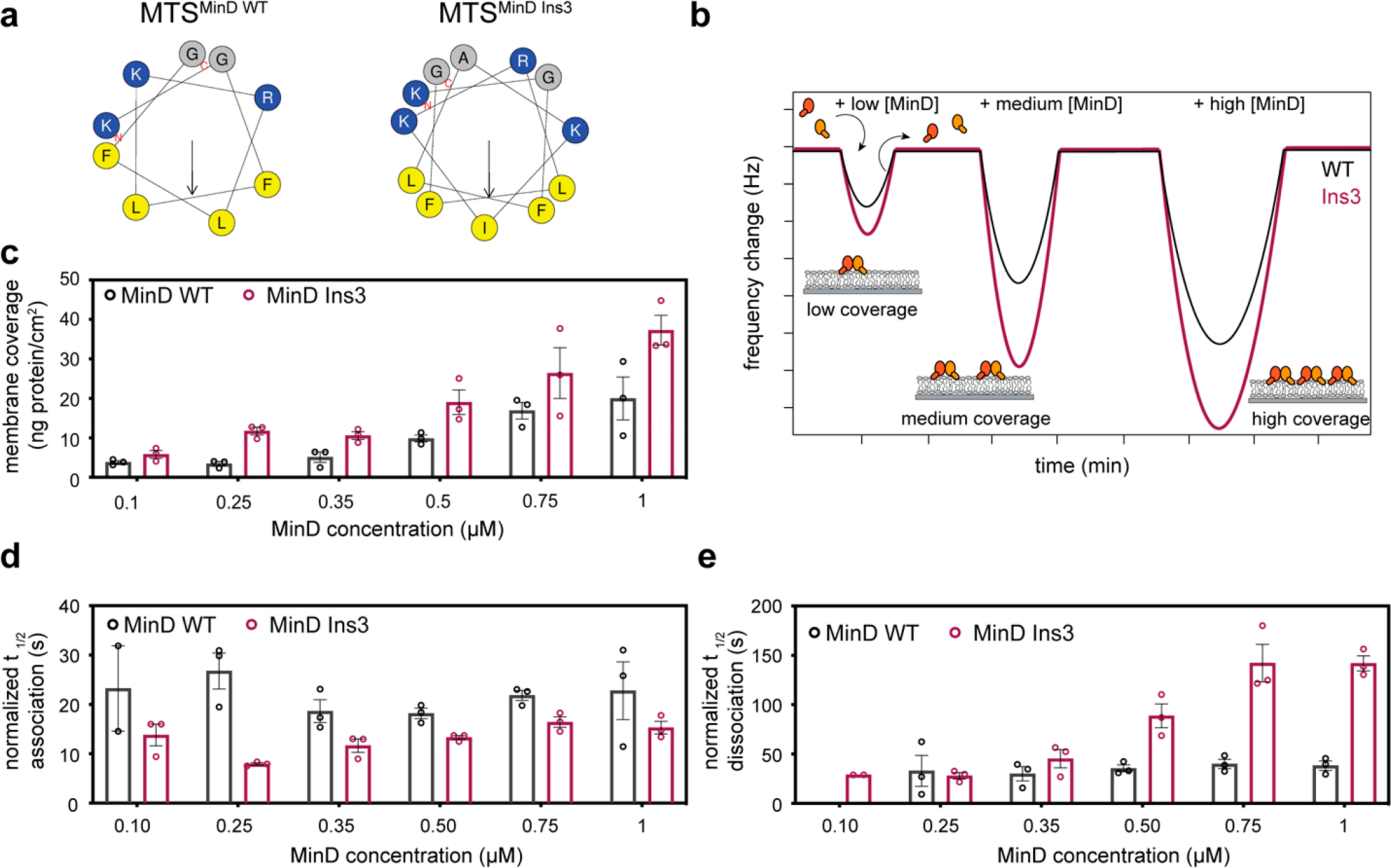
Elongating the MTS increases MinD membrane coverage on flat membranes. (a) Helix wheel representation of the MTS of MinD WT and MinD Ins3 (generated with HELIQUEST^39^). The colors denote small, aliphatic (gray), larger hydrophobic (yellow), and positively charged (blue) amino acids, while the arrow indicates the vector of the hydrophobic moment.^39^ (b) Schematic of a typical QCM experiment, in which the MinD concentration was successively increased between membrane attachment/detachment cycles; (c) membrane coverage obtained from QCM experiments with various concentrations of MinD and MinD Ins3 in the presence of ATP; (d) determined half time (*t*_1/2_) of association (QCM) for the membrane attachment of both MinD WT and MinD Ins3 in the presence of ATP; (e) determined half time of dissociation (QCM) for the membrane detachment of both MinD WT and MinD Ins3 in the presence of ATP. For QCM experiments, three independent experiments were performed each, and bar graphs represent the individual data points with mean values ± SEM.

Cell-free reconstitution has proven particularly enabling for the systematic investigation of the roles of individual components, and their functional modules (Figure 1), in self-organization. This is due to the defined nature of *in vitro* environments, in which factors such as protein concentration or membrane geometry can be precisely controlled. Recent studies based on cell-free reconstitution have revealed important regulatory factors of Min patterns, which include the system’s geometry,^28−30^ salt content, and membrane composition^35^ as well as the concentration and properties of Min proteins.^2,25,26,31−33^ With regard to Min patterns in a simple flat membrane setup, various modulatory parameters have been discovered for MinE, including its membrane interaction,^26,31,32^ conformational switching,^31,34^ and stimulation of MinD’s ATPase activity^31,33^ as well as dimerization and fusion with accessory protein parts.^25,31^ These molecular features can be viewed as functional modules that play defined roles in pattern formation. While MinE’s functional modules have been characterized in considerable detail, MinD has been investigated far less from an engineering perspective. Reverse engineering of MinD is more complicated, due to its central autocatalytic role based on membrane interaction^17^ and ATP hydrolysis^36^ and because of the complexity of its higher-order intermolecular interactions.^19,21^ However, while being more potentially challenging, engineering MinD’s molecular features may also result in a rich phenomenology of Min patterns.

Here, we aim at expanding our understanding of the roles that the Min system’s functional modules play in pattern formation. In particular, we tested how elongating MinD’s MTS via rationally guided mutagenesis affects Min protein patterns *in vitro*. We found that increasing MinD’s membrane affinity via amphipathic residue insertion can give rise to standing wave oscillations on flat membranes, even in the absence of flow or geometric confinement. Moreover, these standing waves promote symmetry breaking and functional gradient formation on time-average, which can be exploited to locally enrich membrane-associated proteins in membrane zones depleted of MinD. Thus, our study provides insights into how distinct patterns and their unique functionalities can be accessed via mutagenesis.

## An Insertion in MinD’s MTS Increases MinD’s Membrane Coverage on Lipid Membranes

MinD’s interaction with the lipid membrane is widely seen as a core requirement for pattern formation.^15^ To confirm this notion, we analyzed membrane binding and self-organization of *E. coli* MinD wild-type (His-MinD/eGFP-MinD WT, as described previously^10,37,38^) and a truncated MinD variant lacking the MTS (Δ256–270, referred to as MinD ΔMTS from here on). We performed quartz crystal microbalance (QCM)^22^ and self-organization^10^ experiments, as both assays are based on a flat membrane geometry, which allows for optimal comparability. From the maximal change in the resonance frequency measured in QCM experiments, we obtained the membrane coverage for MinD WT and the truncation mutant in a nucleotide- and concentration-dependent fashion (Figure 2b, Figure S1a,b). Both, MinD WT and the mutant, showed a slight increase in membrane coverage in the presence of ADP, which may be due to random protein–membrane collisions in the relatively small QCM chambers. Importantly, only the WT showed a strong increase in membrane coverage in the presence of ATP (Figure S1 a, b). Accordingly, while the WT displayed stationary patterns at the tested concentration, the mutant did not appear to bind the membrane or form patterns with MinE-His^25^ in our self-organization assay (Figure S1c), confirming that MinD membrane binding is essential for pattern formation.

To nevertheless explore pattern engineering via changes in MinD’s membrane affinity, we focused on enhancing this feature rather than compromising it. Therefore, we analyzed a MinD insertion mutant termed MinD Ins3, which contains the additional amino acids “AKI” in the MTS and thus an additional turn in the amphipathic membrane targeting sequence^17^ (Figure 2a). This insertion has previously been shown to be capable of interaction with the membrane *in vivo*, indicating intact helicity and amphipathicity.^17^ With the additional turn arising from the “AKI” insertion, the length of MinD Ins3’s engineered MTS resembles the one of *B. subtilis* MinD, which has been shown *in vivo* to support accumulation of a GFP fusion of the MTS even with a single copy of the latter,^16^ indicating an overall higher membrane affinity than the MTS of *E. coli* MinD. Thus, we reasoned that the Ins3 mutant is a suitable variant to dissect the effects of increasing MinD’s membrane affinity on pattern formation.

First, we sought to systematically compare membrane binding between MinD WT and the Ins3 mutant using quartz crystal microbalance (QCM) experiments. In the presence of ATP, MinD Ins3 exhibited a significantly higher membrane coverage than MinD WT across a range of MinD concentrations (Figure 2c), indicating an overall higher membrane affinity of the mutant compared to the WT. In the presence of ADP, we observed weaker membrane interaction for both MinD variants (Figure S2a), as expected for the ADP-bound monovalent state of the MTS when compared to the dimeric ATP-bound state.^15,18^ Nevertheless, the membrane coverage increased with MinD concentration, which was more pronounced for the Ins3 mutant than the WT. At high MinD concentration, MinD Ins3 showed a significantly higher membrane coverage than the WT (Figure S2a), consistent with the report that the MTS of *B. subtilis* MinD, but not the MTS of *E. coli* MinD, supports membrane accumulation of a monomeric GFP-MTS fusion expressed in *E. coli*.^16^

To also assess binding characteristics, we determined the half-times of association and dissociation, that is, the half-time it took the variants to reach the maximal frequency change and return to the baseline, respectively. We opted for these parameters over rate constants obtained from curve fitting, as QCM curves indicated biphasic detachment of MinD from membranes, which was most pronounced for MinD Ins3 in the presence of ATP (Figure S2b). Such biphasic detachment was reminiscent of the concerted detachment of MinD patches, as previously observed by high-speed atomic force microscopy.^21^ Nevertheless, as the molecular basis of this biphasic dissociation in our experiments was not unambiguous, the choice of a particular mathematical model for curve fitting would be highly nontrivial and any results hard to interpret. In contrast, half-times of association/dissociation offer a simple way to compare the WT’s and mutant’s binding and unbinding without any assumptions on the underlying molecular-level processes. Our analysis showed that the Ins3 mutant is characterized by consistently shorter half-times of association in the presence of ATP for all examined MinD concentrations, indicating faster membrane attachment (Figure 2d). In the ADP-state, this effect is also apparent at higher MinD concentrations, whereas no significant difference could be observed at lower concentrations (Figure S2c). Most strikingly, in the presence of ATP, MinD Ins3 dissociates from the membrane significantly slower than the WT with an increasing difference observed at high MinD concentrations (Figure 2e). On the other hand, no consistent trend was apparent for the half-time of dissociation in the presence of ADP (Figure S2d). In sum, in addition to its higher membrane coverage, we found that MinD Ins3 attaches faster and dissociates slower from the membrane compared to MinD WT.

## Formation of Standing Waves on Flat Membranes by MinD Ins3

**Figure 3:**
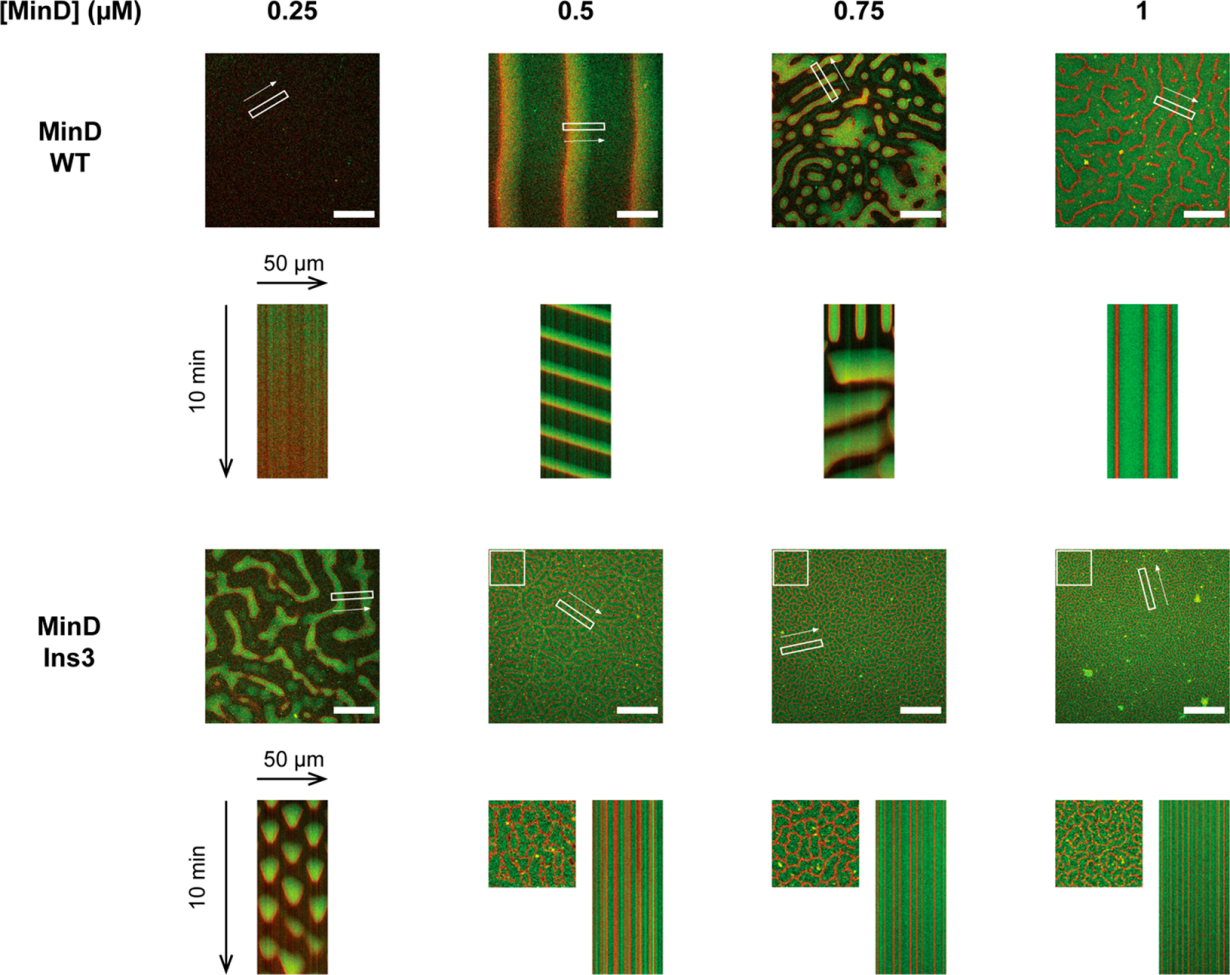
Patterns observed at various concentrations of MinD WT or MinD Ins3 in the presence of MinE-His at a constant concentration of 1 μM. For each condition, mergers of the MinD (green) and MinE (red) channels are shown, along with merged kymographs measured in the rectangular areas in the spatial direction of the arrow. For 0.5–1 μM MinD Ins3, magnifications of the quadratic region indicated in the micrographs are shown left of the kymographs. All micrographs are shown at the start of the kymograph, except for the image corresponding to 0.75 μM MinD WT, which is depicted at a time-point highlighting the coexistence of dynamic and stationary patterns. No pattern was observed for 0.25 μM WT MinD. Protein concentrations: The MinD concentrations indicated in the figure include 20% eGFP-MinD WT or Ins3, respectively, 1 μM MinE-His incl. 10% MinE-KCK-His-Alexa647. Scale bars: 50 μm.

To analyze how enhanced MinD interaction with the lipid membrane impacts pattern formation, we reconstituted MinD WT or the Ins3 mutant together with MinE-His and ATP on supported lipid bilayers. As the relative concentrations of MinD and MinE are known to be an important regulator of pattern formation, we varied the concentration of MinD (including 20% eGFP-MinD), while keeping MinE-His (including 10% MinE-KCK-His-Alexa647) at a fixed concentration. As recently reported for MinE-His,^25^ MinD WT favored traveling waves at lower MinD/MinE ratios, while self-organizing into stationary patterns such as “labyrinths” and spot patterns at higher MinD concentrations (Figure 3). Likewise, as observed previously,^25^ these patterns could also coexist or switch, as exemplified at 750 nM MinD in Figure 3.

We also observed pattern formation when substituting MinD WT with the Ins3 mutant under otherwise identical conditions, demonstrating that self-organization still occurs if MinD’s membrane affinity is increased beyond WT levels (Figure 3). Similar to the WT, we observed stationary patterns at higher MinD concentrations. However, upon reducing the MinD concentration (Figure 3, 250 nM MinD Ins3), MinD Ins3 formed a unique dynamic pattern that coexisted and interconverted with stationary patterns (Figure 3, Figure S3) and was qualitatively distinct from traveling waves. In this type of pattern, Min proteins occupied distinct membrane zones in a switch-like manner. While the detailed appearance varied somewhat in space and time between wave-like and patchy (Figure 3, Figure S3, Movie S1), this type of pattern resulted in spot-like kymographs, which are qualitatively distinct from vertical stripes for stationary patterns and diagonal stripes for traveling waves (Figure 3). On the basis of its similarity to the *in vivo* Min oscillation^40^ and related patterns observed *in vitro*,^26,27^ the mutant pattern can essentially be characterized as a standing-wave oscillation.

To better understand the parameter space in which standing waves form, we focused on the initially identified condition (0.25 μM MinD Ins3) and varied the MinD concentration more gradually around this point, while keeping MinE-His at a constant concentration of 1 μM. Standing waves formed both at slightly lower and higher MinD concentration in our titration from 0.15 to 0.4 μM MinD (Figure S4a). Moreover, at the lower end of tested MinD concentrations, we observed a transition from traveling to standing waves (Figure S4a). In addition, by varying the MinE-His concentration from 0.25 μM to 1 μM at a constant concentration of 0.25 μM MinD, we found that standing waves transitioned to stationary patterns upon reducing the concentration of this MinE variant (Figure S5a). Finally, we asked how variations in absolute MinD concentrations affected standing wave formation, while keeping the MinE/MinD ratio fixed at 4:1. Relative to the initial condition of 0.25 μM MinD Ins3 and 1 μM MinE-His (Figure 3 and Figures S4a, S5a), reducing both protein concentrations by a factor of 2 still allowed for standing wave formation, while stationary patterns formed when they were increased 2-fold (Figure S6a). Taken together, MinD Ins3 and MinE-His tended to form standing waves at rather low absolute and relative MinD concentrations.

We also observed standing waves upon substituting His-MinE for MinE-His (Figures S4b, S5b, S6b, S7). While both MinE-His and His-MinE, formed, and switched with traveling waves for MinD Ins3 at low MinD concentrations, traveling waves appeared more stable for His-MinE (Figure S4b). Furthermore, instead of transitioning from standing waves to stationary patterns upon increasing the MinD concentration, a transition of standing waves back to traveling waves was observed for His-MinE when the concentration of MinD Ins3 was increased (Figure S7). Interestingly, compared to the patterns formed by MinE-His, standing waves formed by His-MinE appeared more robust to changes in relative and absolute protein concentration. In particular, standing waves were also observed with His-MinE for conditions under which MinE-His formed stationary patterns, namely upon lowering the MinE concentration at a fixed MinD concentration (Figure S5b) and for higher absolute concentrations at a conserved MinE/MinD ratio (Figure S6b). These observations are consistent with the general bias toward dynamic as opposed to stationary patterns with the N-terminally tagged MinE variant.^25^

To test if standing waves can be observed more generally for MinD variants with increased membrane affinity, we tested pattern formation with MinD^2xMTS^, which contains two copies of the MTS (MinD^256–270^) separated by a GGS linker. Previously, it was shown that a GFP-2xMTS fusion displays an increased membrane affinity compared to GFP fused to a single copy of the MTS.^16^ Strikingly, upon reconstituting MinD^2xMTS^ with either MinE-His or His-MinE, we observed standing waves for both variants (Figure S8). Thus, while the exact conditions supporting standing waves and alternative patterns vary between specific MinD and MinE variants, we conclude that the formation of standing waves on flat membranes is a general feature of MinD variants with increased membrane affinity.

## Time-Averaged Symmetry Breaking by Standing Waves on Flat Membranes

**Figure 4:**
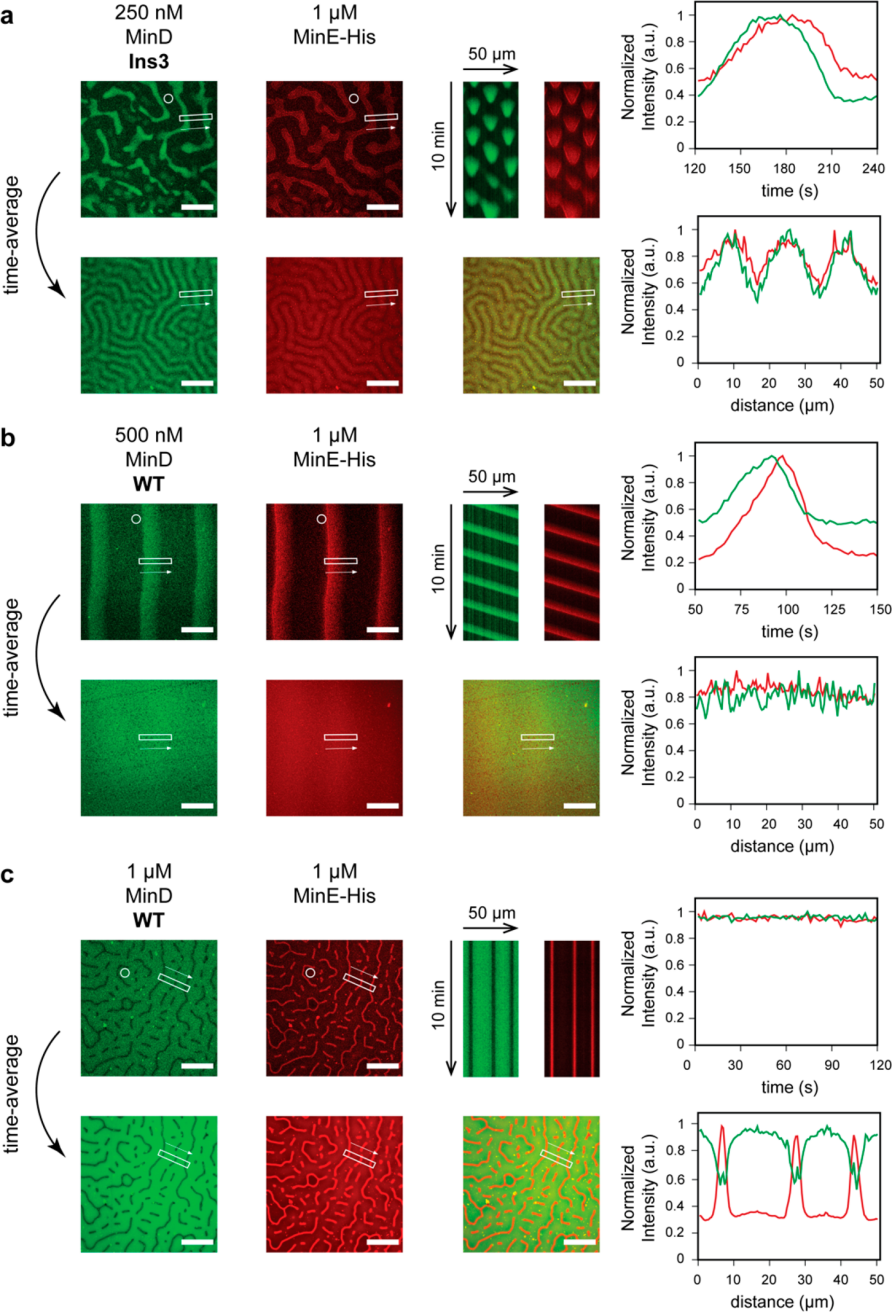
Comparison of different types of patterns, namely, (a) standing waves, (b) traveling waves, and (c) stationary patterns. For each type of pattern, the following is shown: MinD (green) and MinE (red) channels corresponding to the merged micrograph in Figure 3 (top, left), MinD and MinE kymographs measured in the rectangular areas indicated in the micrographs (top, middle), normalized temporal wave profiles for one wave cycle measured in the circular areas indicated in the micrographs (top, right), temporal averages of the MinD and MinE channels and a merge thereof (bottom left), normalized spatial profile of the averaged intensity measured in the same area as the kymographs above (bottom, right). MinD and MinE concentrations stated in the figure include 20% eGFP-MinD WT or Ins3 and 10% MinE-KCK-His-Alexa647, respectively. Scale bars: 50 μm.

To further reveal shared or unique features of the standing waves compared to traveling waves or stationary patterns, we first analyzed temporal profiles of labeled Min proteins within these patterns (Figure 4). Analyzing the temporal profiles of MinD Ins3 and MinE-His for the standing wave oscillations showed that MinE-His followed MinD Ins3, both during membrane attachment and detachment (Figure 4a). This behavior is qualitatively similar to the order of events during traveling wave propagation (Figure 4b). Thus, both, in traveling and standing waves, MinD first accumulates on the membrane and then recruits MinE, which initiates MinD detachment before starting to dissociate itself.^14,21^ As expected, the intensity of MinD and MinE-His did not vary for the stationary patterns within a similar time frame (Figure 4c).

Next, we asked whether the standing wave oscillations emerging with MinD Ins3 can give rise to a key feature of functional Min oscillations *in vivo*, that is, the formation of a time-averaged MinD concentration gradient. While traveling waves generate a largely homogeneous time-averaged MinD distribution (Figure 4b), stationary patterns give rise to a nonhomogeneous distribution that is equivalent to the stationary pattern itself (Figure 4c). In contrast to traveling waves, MinD Ins3’s standing waves yielded a nonhomogenous distribution on the membrane (Figure 4a). Moreover, unlike the equivalent time-averages for the stationary patterns, MinD Ins3 exhibited a time-averaged protein distribution that was distinct from the distribution in any given individual time frame, but instead emerged from the oscillations (Figure 4a). Here, MinD Ins3 was distributed in a pattern with marked maxima at membrane regions occupied by MinD and minima at membrane regions between MinD-occupied patches during standing wave oscillation (Figure 4a), which is best visible when comparing the kymograph with the average intensity within the same region. In this way, the standing wave oscillations emerging with MinD Ins3 bear a striking resemblance to the gradient-forming patterns of Min proteins *in vivo*,^40^ as the oscillations can break the symmetry and give rise to an emergent pattern on time-average.

## Standing-Wave-Directed Enrichment of Downstream Targets on Time-Average

Distributions of proteins in a spatially patterned manner is a desirable property of many synthetic biological systems. Previously, it has been shown that traveling and stationary Min patterns can provide a generic spatial cue for membrane-anchored proteins which dynamically accumulate in membrane regions between MinD-occupied patches.^41−43^ Moreover, it was shown that traveling waves can give rise to a net transport of this cargo,^41^ while stationary patterns give rise to an equally stationary, yet spatially inverted, pattern of these downstream proteins.^25^ The time-averaged symmetry breaking observed with MinD Ins3 suggested that this emergent pattern may be used to localize downstream proteins in a manner that is based on dynamic redistribution but time-averaged local enrichment.

**Figure 5:**
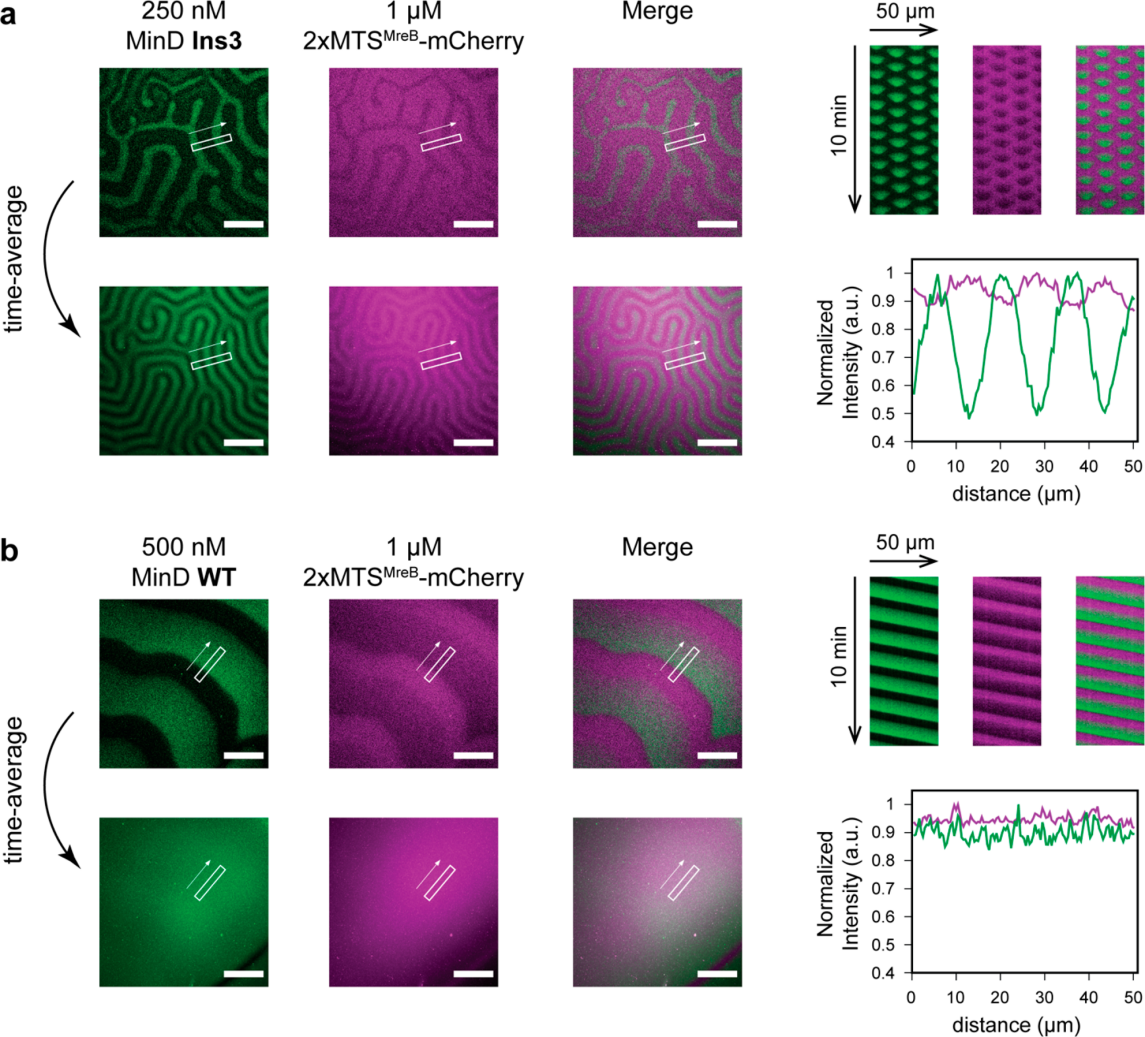
Counter-oscillation and downstream localization of generic peripheral membrane proteins by time-averaged symmetry breaking. For MinD WT or Ins3, the following is shown: MinD (green) and 2xMTS^MreB^-mCherry (magenta) channels and merge thereof (top, left), kymographs of the two channels measured in the rectangular areas indicated in the micrographs and a merge thereof (top, right), temporal averages of the MinD and MinE channels and merge thereof (bottom left), normalized spatial profile of the averaged intensity measured in the same area as the kymographs above (bottom, right). Protein concentrations: The MinD concentrations indicated in the figure include 20% eGFP-MinD WT or Ins3, respectively, 1 μM MinE-His , 1 μM 2xMTS^MreB^-mCherry. Scale bars: 50 μm.

To test such downstream localization, we co-reconstituted MinD Ins3′s standing wave oscillations with a fluorescent protein (mCherry-His) that was fused via its N-terminus to a previously characterized repeat version of the MreB membrane targeting sequence (2xMTS^MreB^)^41^ (Figure 5a). To validate that regulation of downstream proteins occurs under our experimental conditions, we also co-reconstituted 2xMTS^MreB^-mCherry with MinD WT. As expected and discernible from the kymographs, traveling waves gave rise to transport of the membrane-anchored proteins (Figure 5b).

For the Ins3 mutant, kymographs revealed that the 2xMTS^MreB^-mCherry intensity was lowest in the areas occupied by MinD Ins3 at a given time (Figure 5a), indicating a counter-oscillation of the proteins on the membrane. Strikingly, on time-average, this counter-oscillation resulted in a local enrichment of 2xMTS^MreB^-mCherry in the membrane regions in which MinD Ins3 showed minimal occupancy. These results demonstrate that MinD Ins3’s standing waves are capable of transient local depletion of membrane-anchored proteins, which results in unmixing and oscillation-directed local enrichment on time-average.

We have shown that increasing MinD’s membrane affinity via elongation of its MTS enables standing wave oscillations and time-averaged symmetry breaking on flat membranes. Thereby, we have gained a more comprehensive understanding of how biochemical changes in the functional modules of self-organizing proteins can be harnessed to substantially alter the properties of the large-scale protein patterns emerging from their interactions.

Variations of standing waves have previously been observed for the Min system as a result of physical manipulations, such as optical control,^27^ geometric engineering^28,44^ or in a flow cell,^26^ most prominently in the form of “burst” patterns under conditions favoring depletion of MinD.^26^ In contrast, we observed standing waves by biochemical engineering, in a setup consisting only of a flat supported membrane topped with an unperturbed protein reservoir. To the best of our knowledge, this is the first report of this pattern under most reductionistic conditions for reconstituting Min proteins. Comparing the standing waves obtained by mutagenesis with those observed under conditions of protein depletion^26^ and confinement^28,44^ also yields interesting insights into the mechanistic origin of the pattern. First, the increased affinity of an elongated MTS may effectively deplete MinD from the bulk solution compared to MinD WT, due to a higher number of MinD mutant molecules accumulating on the membrane. Second, the standing waves realized by mutagenesis share fundamental similarities to those observed under geometric confinement, including the spot-like appearance in kymographs and the formation of gradient-like zones with low MinD concentration on time average.^29^ In this light, it appears fitting to observe similar patterns upon reducing the ratio of bulk-volume to surface area.^44^

Moreover, the nonhomogeneous protein distribution resulting from the standing waves on time-average can be rendered functional, in terms of locally enriching a downstream protein in the minima of averaged MinD density. While standing waves, stationary patterns,^25^ and traveling waves^41^ can all also be used to locally enrich downstream proteins, time-averaged cargo localization via standing waves may represent the closest equivalent of the *in vivo* Min gradient and could thus be advantageous for harnessing oscillations as spatial cues on flat membranes. Compared to stationary patterns, time-averaged cargo localization could potentially respond to perturbations in the experimental conditions by adaptation of dynamic patterns on a faster time scale. While our results provide a starting point for synthetic biology applications, further engineering of the system’s parts may optimize the performance and address potential challenges. For example, as the observed multistability of different patterns may complicate certain applications, the identification and engineering of conditions suppressing alternative patterns could be an important future research direction. Furthermore, future studies could focus on improving the coherence of standing waves and localization efficiency of downstream cargo. One potential way to realize a higher enrichment of cargo in MinD minima could be to further tune the membrane affinity of both Min proteins and the downstream proteins. Additionally, one could incorporate biochemical inhibitory mechanisms, just like the *in vivo* MinCDE system utilizes MinC’s inhibitory effect on FtsZ assembly.^11^

While theoretical studies had predicted that standing waves may emerge for the Min system^45^*in vitro*, it was unclear how to realize them without the use of external manipulation as mentioned above.^26−28,44^ Our discovery of standing wave oscillations for MinD mutants with elongated MTS provides a tangible route to engineer this pattern solely via mutagenesis. Likewise, theoretical models may now be tested and potentially improved based on our experimental data, which could also reveal generalizable principles underlying the transitions between qualitatively distinct patterns.

Notably, MinD’s MTS has been shown to function as a transplantable protein module to equip unrelated proteins with membrane-associating properties.^16,46^ In this light, future efforts to generate synthetic pattern-forming systems may utilize the MTS and be similarly engineerable via modifying the length of MinD’s MTS. Thus, we hope that our advanced understanding of a pattern-forming protein system with modular features will inform synthetic biology endeavors to forward engineer protein patterns with defined properties.

## Methods

### DNA Constructs

Expression plasmids for MinE-His (pET28a-MinE-His),^25^ MinE-KCK-His (pET28a-MinE-KCK-His),^25^ His-MinE (pET28a-His-MinE),^10,37^ His-KCK-MinE (pET28a-His-KCK-linker-MinE),^25^ His-MinD WT (pET28a-His-MinD_MinE),^10,37^ His-eGFP-MinD WT (pET28a-His-EGFP-MinD),^37,38^ and 2xMTS^MreB^-mCherry-His (pET28a-2xMreBN-mCherry-His)^41^ have been described previously.

Expression plasmids for His-MinD Ins3 and His-eGFP-MinD Ins3 were generated by whole-plasmid PCR amplification of pET28a-His-MinD_MinE and pET28a-His-EGFP-MinD, respectively, with the mutagenic primers MinD-Ins3-FW and MinD-Ins3-RV for both plasmids, followed by digestion of the template DNA with DpnI.

To obtain expression plasmids for His-MinD ΔMTS and eGFP-MinD ΔMTS, coding regions for MinD^1–255^ and EGFP-MinD^2–255^ were amplified from pET28a-His-EGFP-MinD with primers MinD-255Hind3-FW/MinD-255BamHI-RV and MinD-255Hind3-FW/EGFP-BamHI-RV, respectively. The resulting fragments were gel-purified and ligated into pET28a after restriction digest with HinDIII and BamHI and dephosphorylation of the vector. In the case of MinD ΔMTS, the start methionine (MinD^residue 1^) was further removed via PCR-based linearization of the previous step’s ligation product using the primer pair pET28aMinDlin-FW/pET28aMinDlin-RV, followed by digestion of the template with DpnI, gel purification, and religation.

To obtain expression plasmids for MinD^2xMTS^ and eGFP-MinD^2xMTS^, pET28a-His-MinD_MinE and pET28a-His-EGFP-MinD were linearized via PCR with primer pairs linMinDMTS-FW/linMinDMTS-RV and linEGFPMinDMTS-1/linEGFPMinDMTS-2, respectively, which was followed by DpnI digestion and gel purification of the PCR products. Inserts were amplified from a pEX-A2 vector containing a synthetic insert sequence (ordered from Eurofins Scientific, Luxembourg) with primer pairs 2xMTS-FW/2xMTS-RV for MinD^2xMTS^ and MTSminD-FW/T7term-RV for eGFP-MinD^2xMTS^, respectively, followed by gel purification. Plasmids were then assembled from linearized vector and amplified inserts via GeneArt Seamless Cloning and Assembly (Thermo Fisher Scientific, Waltham, MA, USA) or the NEBuilder HiFi DNA Assembly Master Mix (NEB, Ipswich, MA, USA) according to the manufacturers’ instructions. Primer and insert sequences are given in Table S1 in the Supporting Information.

### Protein Purification and Labeling

WT and mutant MinD and MinE variants as well as 2xMTS^MreB^-mCherry-His were expressed and purified as described previously.^10,25,37,41^ Protein concentrations were measured via Bradford assay. MinE-KCK-His and His-KCK-MinE were labeled with Alexa Fluor 647 according to the manufacturer’s instructions, and unbound dye was removed as described previously.^25^

For simplicity, we refer to His-MinD and His-eGFP-MinD WT and mutant proteins as well as 2xMTS^MreB^-mCherry-His simply as MinD, eGFP-MinD, and 2xMTS^MreB^-mCherry despite their His-tags. However, we explicitly refer to MinE-His or His-MinE, as we report experiments with both variants, which were previously shown to act differently on Min patterns.^25^

### QCM Experiments

Prior to each measurement, silicon dioxide (SiO_2_)-coated quartz crystal sensors (Biolin Scientific, Gothenburg, Sweden) were treated with a 3:1 mixture of sulfuric acid and hydrogen peroxide (piranha-solution). Subsequently, sensors were rinsed with ultrapure water, dried under a stream of nitrogen, and mounted in the flow modules of the QSense Analyzer (Biolin Scientific, Gothenburg, Sweden). Resonance frequencies were obtained for both air and buffer (QSoft Version 2.5.36; Biolin Scientific, Gothenburg, Sweden). According to the equation specified in Cho et al.,^47^ second signature S2 values were determined to qualitatively assess the systems performance (Table S2). After baseline stabilization, supported lipid bilayer formation (SLB) was induced through constant injection (flow rate: 0.15 mL/min) of a 1 mg/mL mixture of small unilamellar vesicles (DOPC/DOPC, 70:30 mol %) in Min Buffer (25 mM Tris-HCl pH 7.5, 150 mM KCl, 5 mM MgCl_2_), spiked with 5 mM CaCl_2_. After lipid deposition, 0.1, 0.25, 0.35, 0.5, 0.75, and 1 μM of MinD or MinD Ins3 with either 2.5 mM ATP or ADP were adsorbed and desorbed under constant flow (0.1 mL/min). All presented and analyzed data sets correspond to the frequency changes of the ninth overtone, and measurements were conducted at 24 °C. Raw data export was performed using QTools 3 version 3.1.25.604 (Biolin Scientific, Gothenburg, Sweden). Further data analysis was executed using a customized MATLAB R2018a (The MathWorks, Inc., Natick, USA) script to extract the frequency change for each individual binding event. Membrane coverage was then calculated according to the Sauerbrey equation,^48^ and data visualization was performed using GraphPad Prism 7.0d (GraphPad Software, La Jolla, USA).

### Preparation of Supported Lipid Bilayers for Self-Organization Assays

Coverslips were cleaned with Piranha solution, and assay chambers were prepared as described previously.^25,37^ Small unilamellar vesicles (SUVs) were prepared as follows. DOPC and DOPG (both from Avanti Polar Lipids, Alabaster, USA) were dissolved at 25 mg/mL in chloroform and mixed in a molar DOPC/DOPG ratio of 70:30. The lipid mixture in a 1.5 mL glass vial was then dried under a constant stream of nitrogen and vacuum applied for at least 30 min. The dried lipid film at the rim of the vial was then rehydrated with Min buffer (see QCM section) at 4 mg/mL by vortexing every 20 min while being incubated for 1 h at 37 °C. The vesicles were then sonicated until clear in a water bath sonifier. SUVs were stored as 20 μL aliquots at −20 °C until further use and briefly sonicated before such. SLBs were formed in the assay chambers with the prepared SUVs as described by Glock et al.^25^

### Self-Organization Assays

Self-organization assays were performed in assay chambers with SLB at the bottom, topped with assay components in Min buffer (200 μL final assay volume).^10,37^ Proteins were added to Min buffer at the concentrations specified in the figure captions along with 2.5 mM ATP (F. Hoffmann-La Roche, Basel, Switzerland), and the solution was carefully mixed by pipetting.

### Microscopy and Image Processing

Confocal fluorescence imaging was carried out with a Zeiss LSM 780 or LSM 800 laser scanning microscope using a Zeiss C-Apochromat 40*x*/1.20 water-immersion objective (Carl Zeiss, Oberkochen, Germany). Laser intensities and gains were adjusted for optimal imaging of the individual samples. Images were analyzed and adjusted for brightness and contrast using Fiji.^49^ Plotted intensities were normalized to the maximum value for the analyzed fluorophore in any given plot.

## Supporting Information Available

DNA sequences relevant for plasmid construction; additional microscopy data for MinD WT and Ins3 in the presence of MinE-His or His-MinE at various protein concentrations; additional figures that support the text (PDF)
Standing wave pattern occurring at 250 nM MinD Ins3 in the presence of MinE-His (AVI)
Coexisting stationary and dynamic patterns occurring at 250 nM MinD Ins3 in the presence of MinE-His (AVI)
Standing wave pattern occurring at 250 nM MinD Ins3 in the presence of His-MinE(AVI)
Counter-oscillation of MinD Ins3 and a peripheral membrane protein (AVI)
Plasmid maps corresponding to expression plasmids for MinD Ins3, MinD^2xMTS^, eGFP-MinD Ins3 and eGFP-MinD^2xMTS^ (ZIP)

## Supplementary Material

sb0c00604_si_001.pdf

sb0c00604_si_002.avi

sb0c00604_si_003.avi

sb0c00604_si_004.avi

sb0c00604_si_005.avi

## Abbreviations

ATP: adenosine triphosphate
DOPC: 1,2-dioleoyl-*sn*-glycero-3-phosphocholine
DOPG: 1,2-dioleoyl-*sn*-glycero-3-phospho-(1′-rac-glycerol)
MTS: membrane targeting sequence
QCM: quarz crystal microbalance
SEM: standard error of the mean
SLB: supported lipid bilayer
SUV: small unilamellar vesicle
WT: wild-type
