# Supplementary material for: Increasing MinD’s Membrane Affinity Yields Standing Wave Oscillations and Functional Gradients on Flat Membranes
Source: ACS Synth Biol. 2021 Apr 21;10(5):939–49. doi: 10.1021/acssynbio.0c00604 (PMC8155659; doi:10.1021/acssynbio.0c00604)
Supplement: Supplementary file 1 — sb0c00604_si_001.pdf [file sb0c00604_si_001.pdf]

Increasing MinD's membrane affinity yields standing wave oscillations and functional gradients on flat membranes

Simon Kretschmer[1,2], Tamara Heermann[1], Andrea Tassinari[1], Philipp Glock[1], and Petra Schwille[*,1]

1 Department of Cellular and Molecular Biophysics, Max-Planck-Institute of Biochemistry, Am Klopferspitz 18, 82152 Martinsried, Germany
2 Current affiliation: Department of Bioengineering and Therapeutic Sciences, University of California San Francisco, San Francisco, California 94158, United States of America

* Corresponding Author; E-Mail: schwille@biochem.mpg.de

This PDF file includes:
Figs S1 to S8
Tables S1 to S2
Captions for movies S1 to S4

Supplementary Figures

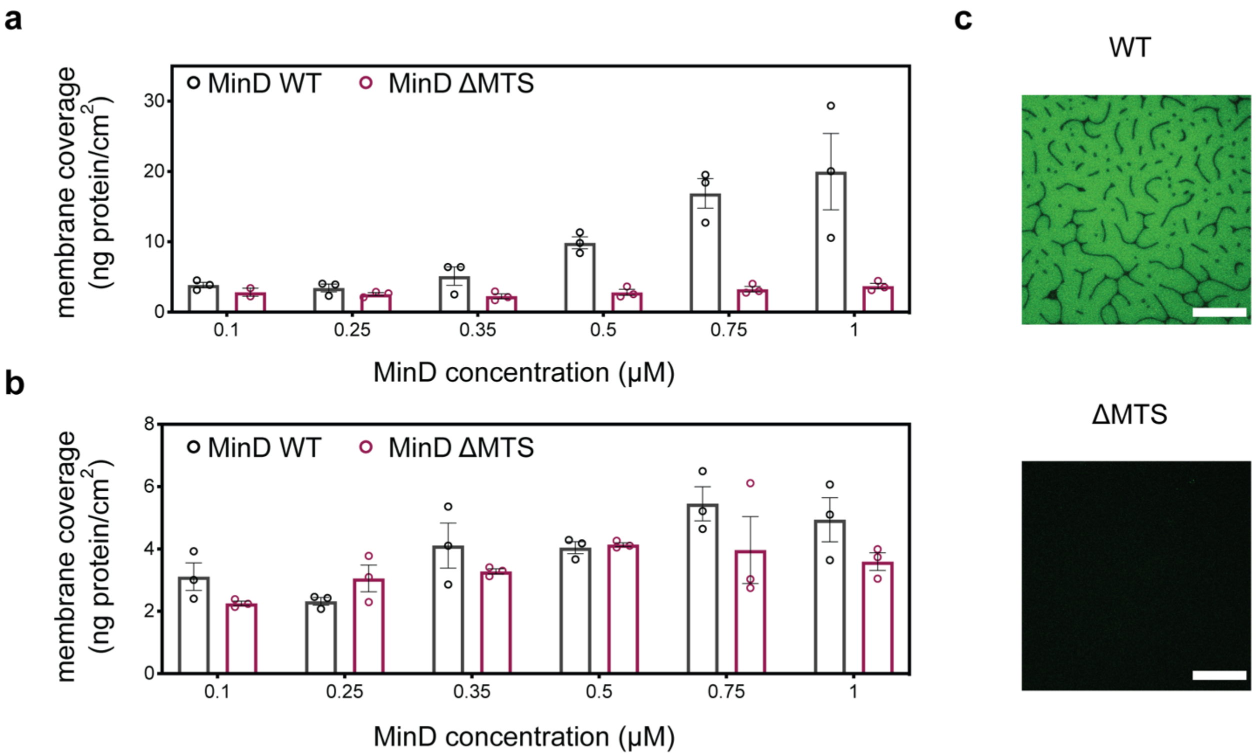

Figure S1. MinD's MTS is essential for pattern formation. **a) & b)** Membrane coverage obtained from QCM experiments with various concentrations of MinD WT and MinD ΔMTS in the presence of **a)** ATP or **b)** ADP. All QCM experiments were performed in triplicates and bar graphs represent individual data points and the determined mean values ± SEM. **c)** Confocal micrographs from self-organization assays with 1 μM MinD WT or ΔMTS (incl. 20% eGFP-MinD WT or ΔMTS) and 1 μM MinE-His. Scale bars: 50 μm.

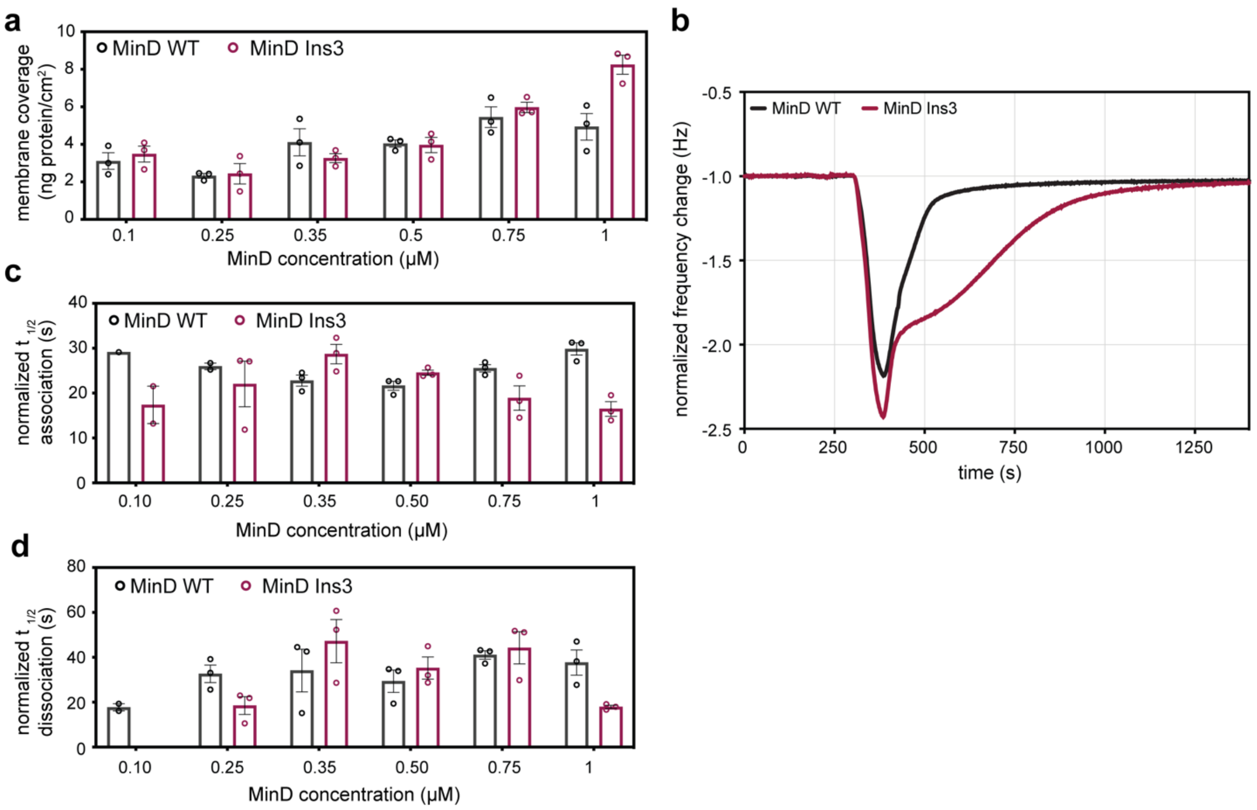

Figure S2. Elongating the MTS alters MinD's membrane attachment and detachment properties. **a)** Determined membrane coverage of MinD WT or MinD Ins3 in the presence of ADP for increasing protein concentrations. **b)** Representative frequency change determined for the addition of 1 μM protein MinD (black line) or MinD Ins3 (magenta line) respectively, displaying the biphasic dissociation of both constructs from the lipid interface. **c)** half time ($t_{1/2}$) of membrane association of MinD and MinD Ins3 with ADP. **d)** half time of membrane dissociation of MinD and MinD Ins3 in the presence of ADP. All displayed QCM experiments were at least performed in triplicates and bar graphs represent individual data points and the determined mean values ± SEM.

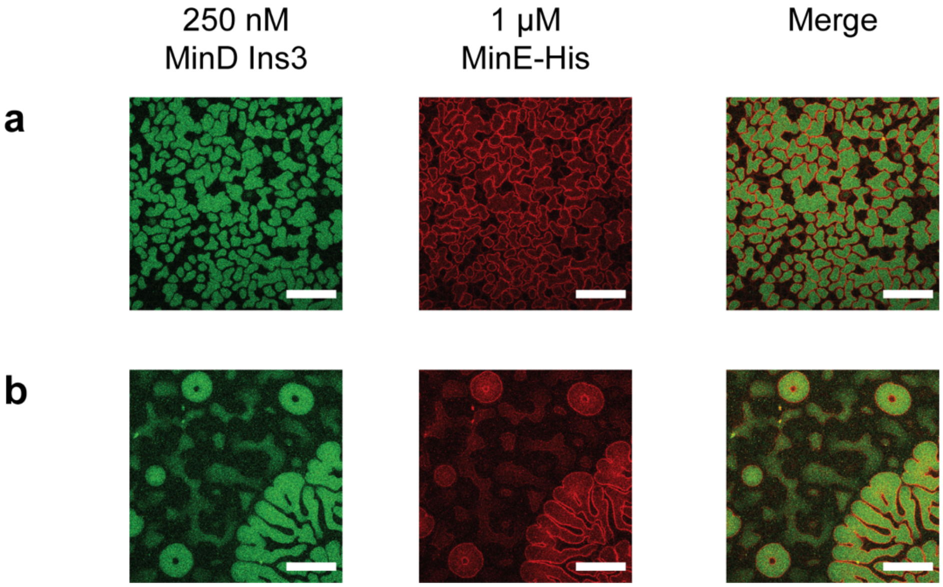

Figure S3. Alternative patterns observed with MinD Ins3 under the same experimental conditions as standing waves: **a)** Example of a sample region with a spot-like pattern, **b)** Example of a sample region with co-existence of dynamic patterns and comparatively stationary patterns (see also Supplementary Movie S2). The shown images were acquired in the same sample as the standing waves in Fig. 3 and Fig. 4. Protein concentrations: 250 nM MinD Ins3 incl. 20% eGFP-MinD Ins3, 1 µM MinE-His incl. 10% MinE-KCK-His-Alexa647. Scale bars: 50 µm.

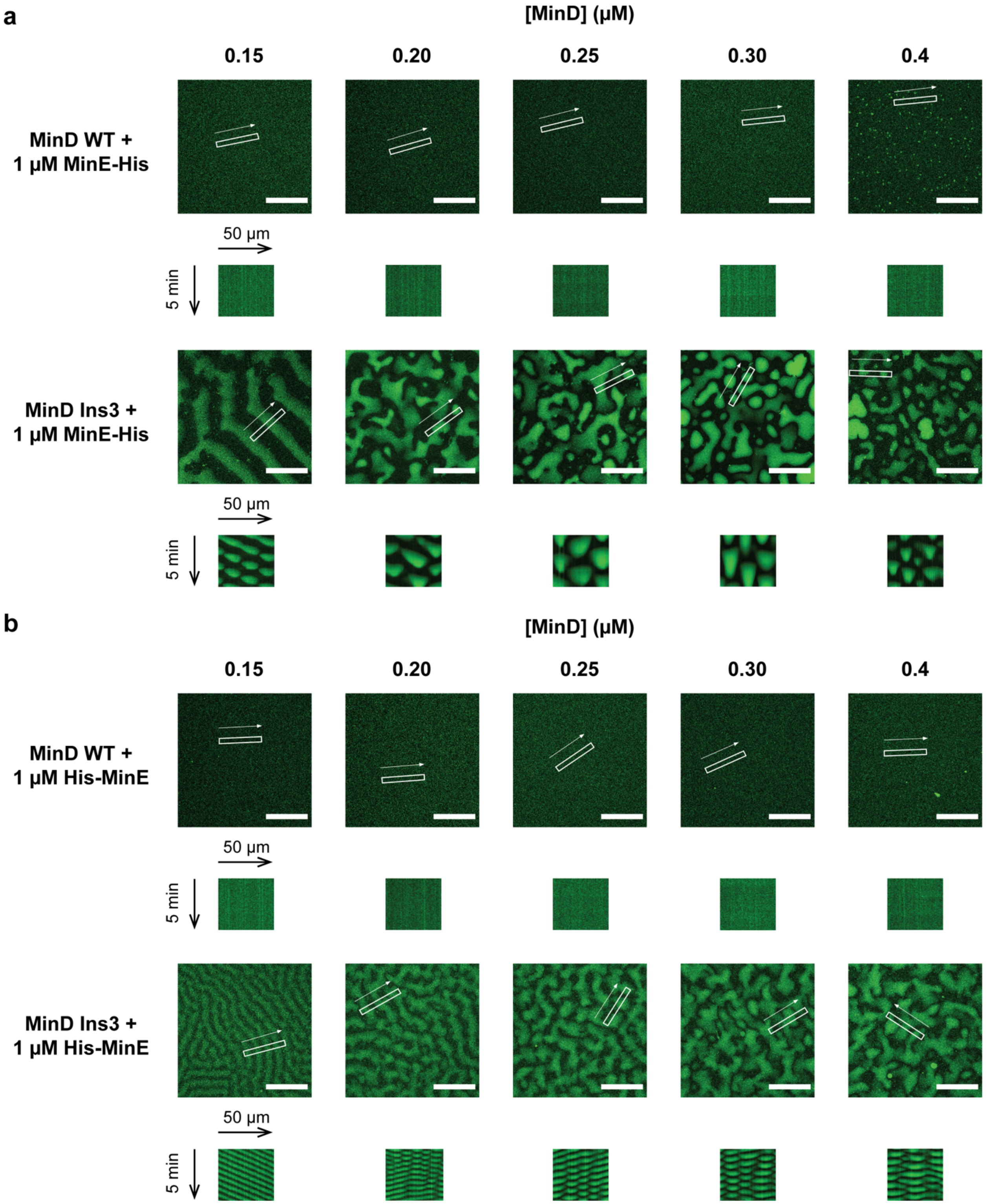

Figure S4. Patterns observed at concentrations between 0.15 and 0.4 µM MinD WT or MinD Ins3 in the presence of **a)** MinE-His, or **b)** His-MinE, at a constant MinE concentration of 1 µM. MinD

channels and kymographs measured in the indicated rectangular areas are shown. Indicated MinD concentrations include 20% eGFP-MinD WT or Ins3 respectively. Scale bars: 50 μm.

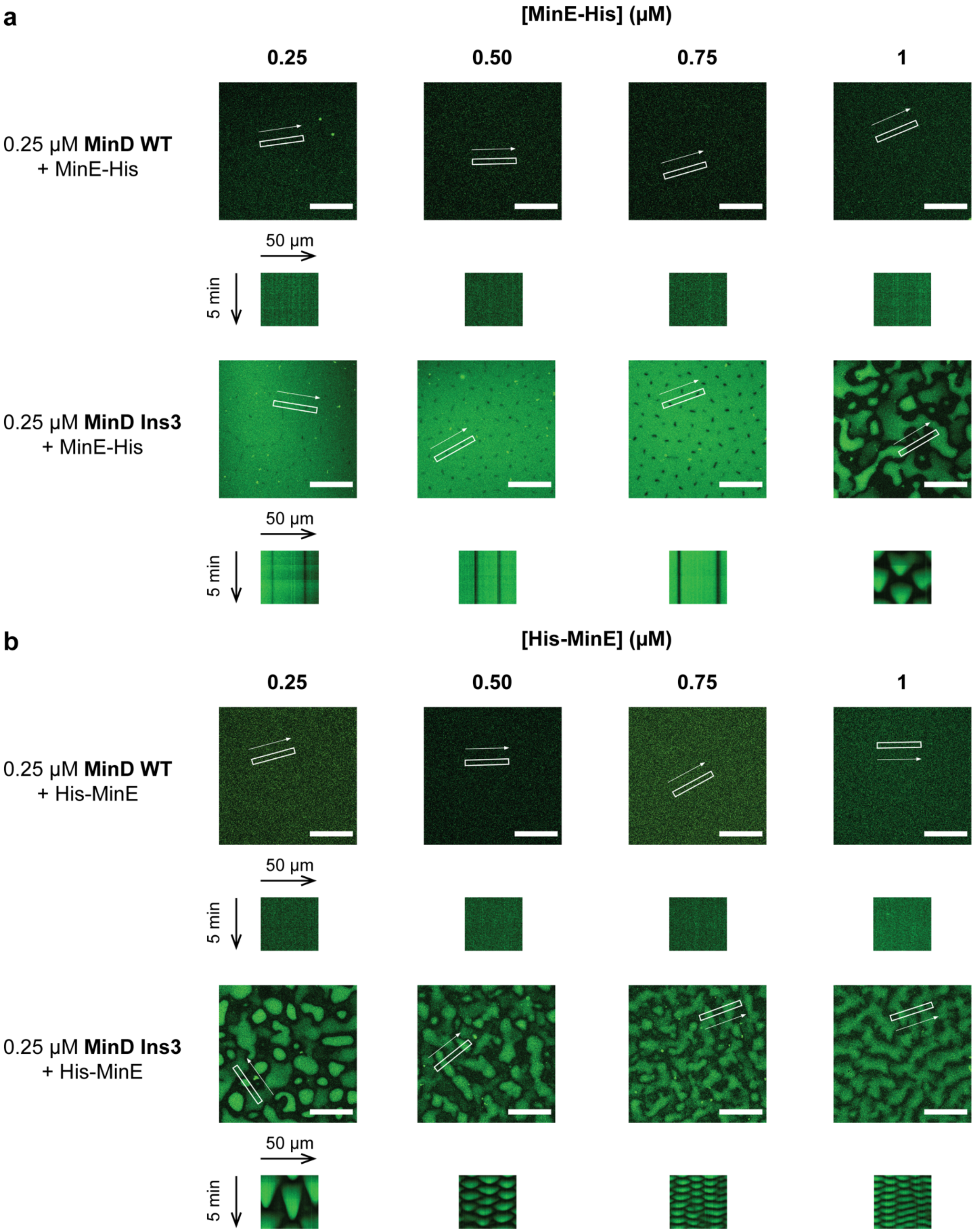

Figure S5. Patterns observed for MinD WT or MinD Ins3, at a constant MinD concentration of 0.25 μM, in the presence of varying concentrations of **a)** MinE-His, or **b)** His-MinE. MinD

channels and kymographs measured in the indicated rectangular areas are shown. No patterns were observed for MinD WT at any of the concentrations examined here. Indicated MinD concentrations include 20% eGFP-MinD WT or Ins3 respectively. Scale bars: 50 μm.

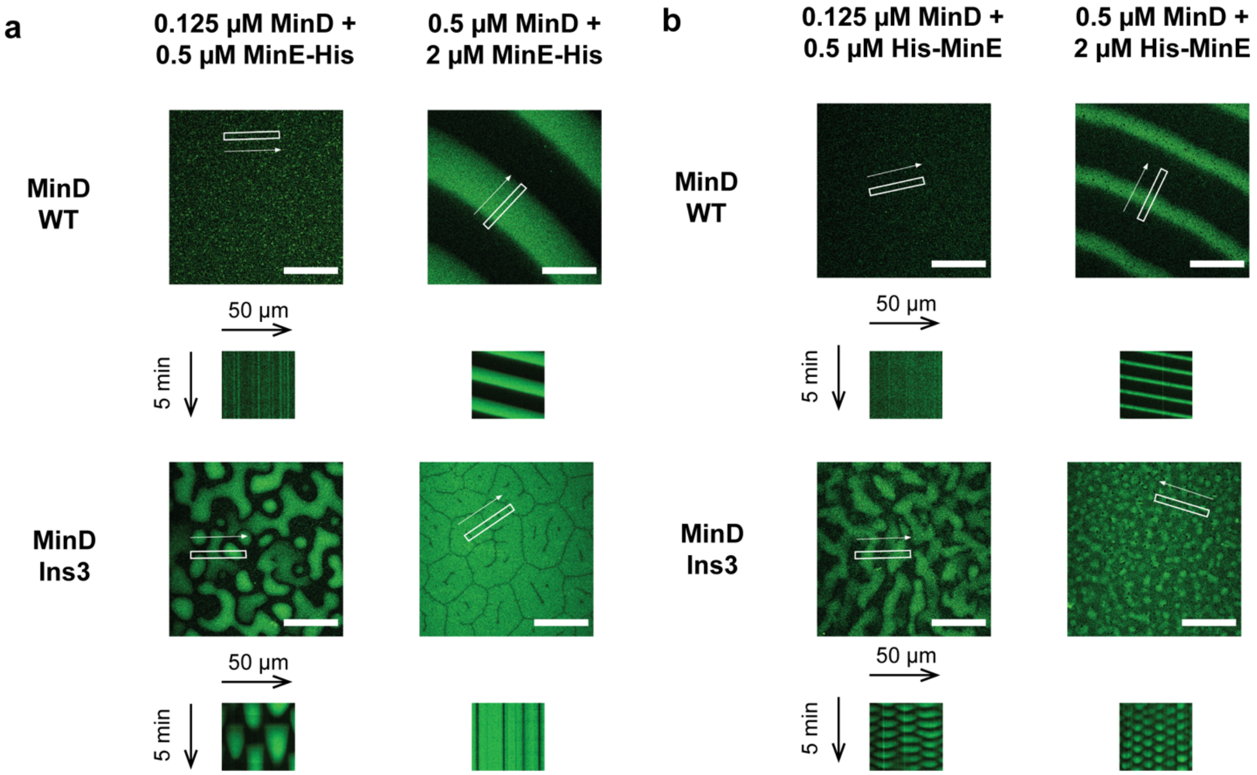

Figure S6. Patterns observed at different absolute MinD and MinE concentrations but a constant MinE/MinD ratio of 4:1 for MinD WT or MinD Ins3 in the presence of **a)** MinE-His, or **b)** His-MinE. MinD channels and kymographs measured in the indicated rectangular areas are shown. No patterns were observed for MinD WT at 0.125 µM MinD + 0.5 µM MinE-His/His-MinE. Indicated MinD concentrations include 20% eGFP-MinD WT or Ins3 respectively. Scale bars: 50 µm.

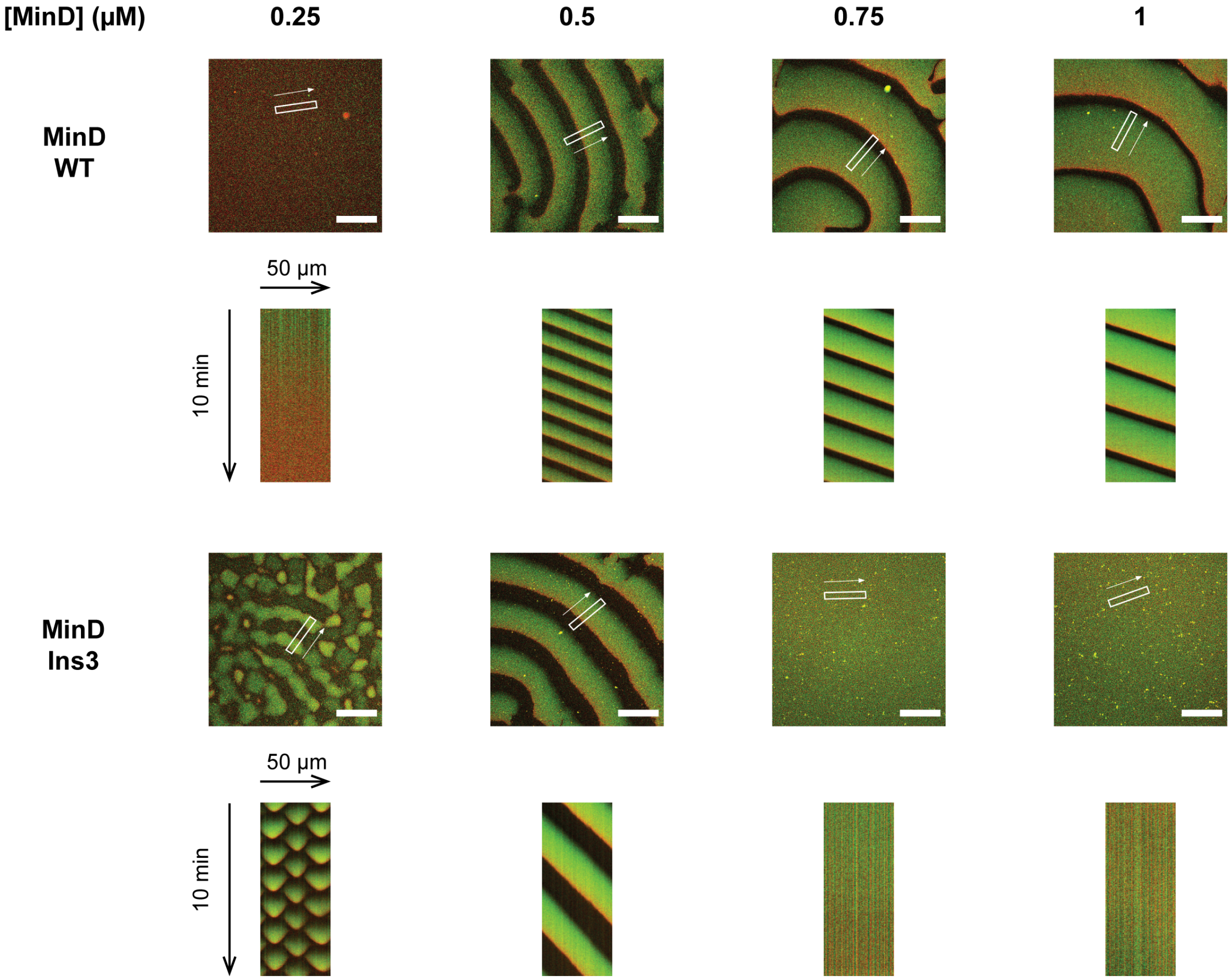

Figure S7. Patterns observed at various concentrations of MinD WT or MinD Ins3 in the presence of His-MinE at a constant concentration of 1 µM. Merges of the MinD (green) and MinE (red) channels and merged kymographs measured in the indicated areas are shown. The MinD concentrations indicated in the figure include 20% eGFP-MinD WT or Ins3 respectively, 1 µM His-MinE incl. 10% Alexa647-His-KCK-MinE. Scale bars: 50 µm.

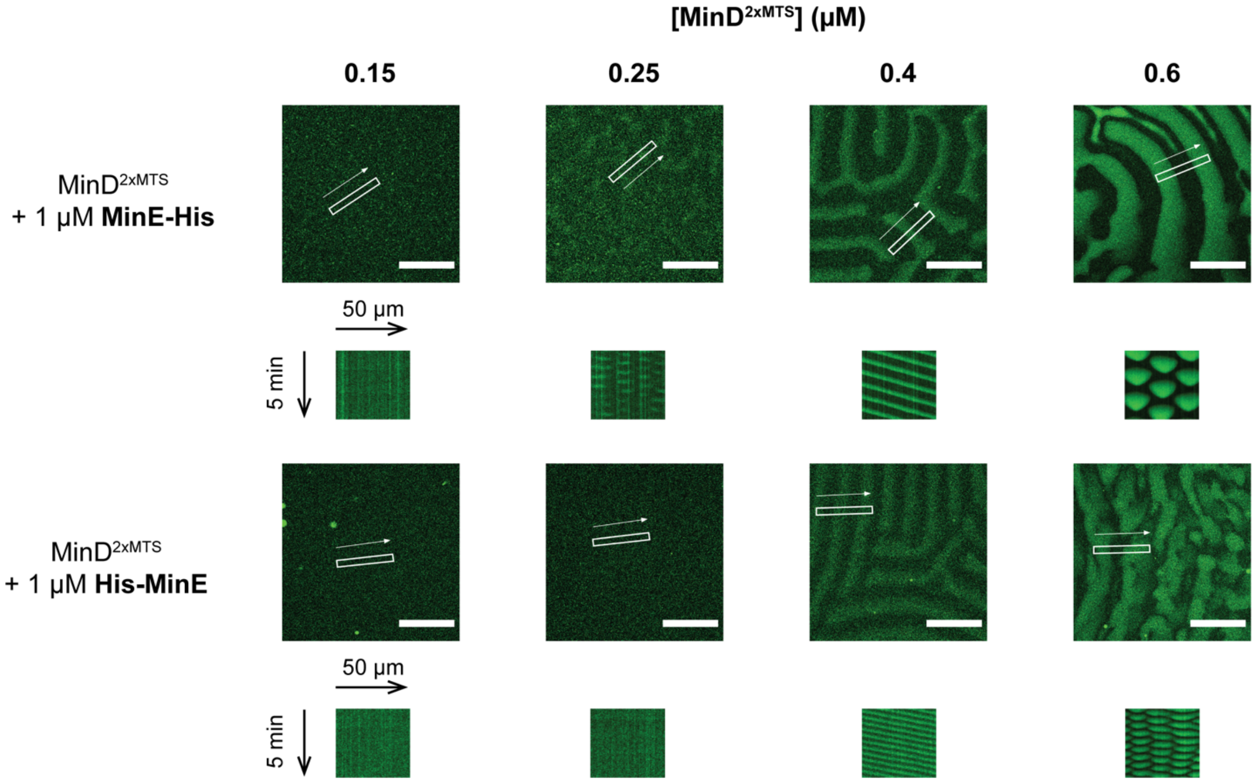

Figure S8. Patterns observed at different concentrations of MinD^{2xMTS} in the presence of 1µM MinE-His or 1 µM His-MinE. MinD channels and kymographs measured in the indicated rectangular areas are shown. No patterns were observed for 0.15 µM MinD^{2xMTS} + 1µM MinE-His/His-MinE as well as 0.25 µM MinD^{2xMTS} + 1 µM His-MinE. Indicated MinD concentrations include 20% eGFP-MinD WT or Ins3 respectively. Scale bars: 50 µm.

Supplementary Tables

Table S1. Primers and MTS insert sequences used in this study

MinD-Ins3-FW	AGAAGAAAGGCTTCCTCGCGAAAATTAAACGCTTGTTCGGAGG
MinD-Ins3-RV	CCTCCGAACAAGCGTTTAATTTTCGCGAGGAAGCCTTTCTTCT
MinD-255Hind3-FW	CTAAAGCTTATTAGAAGCGGAAAGGACGTTCTT
MinD-255BamHI-RV	CTAGGATCCATGGCACGCATTATTGTTGTTACTTC
EGFP-BamHI-RV	CTAGGATCCATGGTGAGCAAGGGCGAGGAG
pET28aMinDlin-FW	GGATCCGCGACCCATTTG
pET28aMinDlin-RV	GCACGCATTATTGTTGTTACTTCG
linMinDMTS-FW	GAAGCGGAAAGGACGTTC
linMinDMTS-RV	ATTGAAGAAGAGAAGAAAGGCT
2xMTS-FW	GCCTTTCTTCTCTTCTTCAATAG
2xMTS-RV	GAAGAACGTCCTTTCCGC
linEGFPMinDMTS-1	GGGGCCTCTAAACGGG
linEGFPMinDMTS-2	CTCCGAACAAGCGTTT
T7term-RV	CCGTTTAGAGGCCCCAAG
MTSminD-FW	CAAACGCTTGTTCGGAGG
Amplified insert sequence from pEX_A2 synthetic construct for MinD^{2xMTS}	GCCTTTCTTCTCTTCTTCAATAGATCCACCTCCTCCGAACAAGCGTTTGAGGAAGCCTTTCTTCTCTTCTTCAATGAAGCGGAAAGGACGTTCTTC
Amplified insert sequence from pEX_A2 synthetic construct for eGFP-MinD^{2xMTS}	CCGTTTAGAGGCCCCAAGGGGTTATGCTAGTTATTGCTCAGCGGTGGCAGCAGCCAACTCAGCTTCCTTTCGGGCTTTGTTAGCAGCCGGATCTCAGTGGTGGTGGTGGTGGTGCTCGAGTGCGGCCGCAAGCTTTATCCTCCGAACAAGCGTTTGAGGAAGCCTTTCTTCTCTTCTTCAATAGATCCACCTCCTCCGAACAAGCGTTTG

Table S2. Second signature (S_2) values for the evaluation of the QCM system performance during the analysis of MinD WT and MinDIns3 membrane coverage with either ATP or ADP. S2 values are indicated and the deviation from the reference values for harmonics 3, 5, 7, 9, 11 and 13 (2.45, 2.5, 2.49, 2.45, 2.46 and 2.47, respectively) is specified in percent[1].

		**overtone**	**replicate 1**		**replicate 2**		**replicate 3**	
			**S_2**	**deviation [%]**	**S_2**	**deviation [%]**	**S_2**	**deviation [%]**
**ATP**	**MinD WT**	1	2,47	-	2,22	-	2,26	-
		3	2,46	0,01	2,43	0,01	2,45	0,00
		5	2,51	0,00	2,43	0,03	2,41	0,05
		7	2,51	0,01	2,47	0,01	2,47	0,01
		9	2,50	0,03	2,43	0,01	2,43	0,01
		11	2,54	0,04	2,50	0,02	2,48	0,01
		13	2,55	0,04	2,57	0,05	2,50	0,02
	**MinD Ins3**	1	2,20	-	2,29	-	2,32	-
		3	2,47	0,01	2,44	0,01	2,50	0,02
		5	2,47	0,01	2,40	0,05	2,47	0,02
		7	2,51	0,01	2,49	0,00	2,51	0,01
		9	2,48	0,01	2,44	0,00	2,52	0,03
		11	2,51	0,03	2,50	0,02	2,62	0,08
		13	2,54	0,04	2,54	0,04	2,75	0,14
**ADP**	**MinD WT**	1	2,15	-	2,27	-	13,57	-
		3	2,45	0,00	2,43	0,01	4,57	1,06
		5	2,45	0,02	2,40	0,05	3,26	0,38
		7	2,50	0,00	2,43	0,03	2,93	0,22
		9	2,46	0,01	2,45	0,00	2,75	0,15
		11	2,49	0,01	2,46	0,00	2,64	0,09
		13	2,52	0,03	2,02	0,22	2,52	0,03
	**MinD Ins3**	1	2,29	-	2,28	-	2,25	-
		3	2,44	0,00	2,42	0,02	2,46	0,00
		5	2,39	0,06	2,43	0,03	2,44	0,03
		7	2,43	0,03	2,44	0,02	2,48	0,00
		9	2,44	0,00	2,57	0,06	2,45	0,00
		11	2,45	0,00	2,46	0,00	2,51	0,02
		13	2,49	0,01	2,54	0,04	2,56	0,04

Supplementary Movies

Supplementary Movie S1. Standing wave pattern occurring at 250 nM MinD Ins3 in the presence of MinE-His. The movie shows the merged MinD and MinE channels. Protein concentrations: 250 nM MinD Ins3 incl. 20% eGFP-MinD Ins3, 1 µM MinE-His incl. 10% MinE-KCK-His-Alexa647. Scale Bar: 50 µm.

Supplementary Movie S2. Co-existing stationary and dynamic patterns occurring at 250 nM MinD Ins3 in the presence of MinE-His. The movie shows the merged MinD and MinE channels. Protein concentrations: 250 nM MinD Ins3 incl. 20% eGFP-MinD Ins3, 1 µM MinE-His incl. 10% MinE-KCK-His-Alexa647. Scale Bar: 50 µm.

Supplementary Movie S3. Standing wave pattern occurring at 250 nM MinD Ins3 in the presence of His-MinE. The movie shows the merged MinD and MinE channels. Protein concentrations: 250 nM MinD Ins3 incl. 20% eGFP-MinD Ins3, 1 µM His-MinE incl. 10% Alexa647-His-KCK-MinE. Scale Bar: 50 µm.

Supplementary Movie S4. Counter-oscillation of MinD Ins3 and a peripheral membrane protein. From left to right, the movie shows the eGFP-MinD Ins3, 2xMTS^{MreB}-mCherry and merged channels. Protein concentrations: 250 nM MinD Ins3 incl. 20% eGFP-MinD Ins3, 1 µM MinE-His, 1 µM 2xMTS^{MreB}-mCherry-His. Scale Bar: 50 µm.

References

[1] Cho, N. J., Frank, C. W., Kasemo, B., and Hook, F. (2010) Quartz crystal microbalance with dissipation monitoring of supported lipid bilayers on various substrates, *Nat Protoc 5*, 1096-1106.
